# Delayed Onset Muscle Soreness (DOMS): The Repeated Bout Effect and Chemotherapy-Induced Axonopathy May Help Explain the Dying-Back Mechanism in Amyotrophic Lateral Sclerosis and Other Neurodegenerative Diseases

**DOI:** 10.3390/brainsci11010108

**Published:** 2021-01-15

**Authors:** Balázs Sonkodi

**Affiliations:** Department of Health Sciences and Sport Medicine, University of Physical Education, Alkotas u. 44, H-1123 Budapest, Hungary; bsonkodi@gmail.com

**Keywords:** amyotrophic lateral sclerosis, delayed onset muscle soreness, repeated bout effect, peripheral sensory axonopathy, proprioception, eccentric contraction, NMDA receptor

## Abstract

Delayed onset muscle soreness (DOMS) is hypothesized to be caused by glutamate excitotoxicity-induced acute compression axonopathy of the sensory afferents in the muscle spindle. Degeneration of the same sensory afferents is implicated in the disease onset and progression of amyotrophic lateral sclerosis (ALS). A series of “silent” acute compression proprioceptive axonopathies with underlying genetic/environmental factors, damaging eccentric contractions and the non-resolving neuroinflammatory process of aging could lead to ALS disease progression. Since the sensory terminals in the muscle spindle could not regenerate from the micro-damage in ALS, unlike in DOMS, the induced protective microcircuits and their long-term functional plasticity (the equivalent of the repeated bout effect in DOMS) will be dysfunctional. The acute stress invoking osteocalcin, bradykinin, COX1, COX2, GDNF, PGE2, NGF, glutamate and N-methyl-D-aspartate (NMDA) receptors are suggested to be the critical signalers of this theory. The repeated bout effect of DOMS and the dysfunctional microcircuits in ALS are suggested to involve several dimensions of memory and learning, like pain memory, inflammation, working and episodic memory. The spatial encoding of these memory dimensions is compromised in ALS due to blunt position sense from the degenerating proprioceptive axon terminals of the affected muscle spindles. Dysfunctional microcircuits progressively and irreversibly interfere with postural control, with motor command and locomotor circuits, deplete the neuroenergetic system, and ultimately interfere with life-sustaining central pattern generators in ALS. The activated NMDA receptor is suggested to serve the “gate control” function in DOMS and ALS in line with the gate control theory of pain. Circumvention of muscle spindle-loading could be a choice of exercise therapy in muscle spindle-affected neurodegenerative diseases.

## 1. Introduction

Delayed onset muscle soreness (DOMS) is defined by delayed onset soreness, muscle stiffness, loss of force-generating capacity, reduced joint range of motion and decreased proprioceptive function [1]. Amyotrophic lateral sclerosis (ALS) is an adult-onset lethal multifactorial, multisystem neurodegenerative disease that causes paralysis through the progressive death of motoneurons (MNs) [2]. Sensory deficits and impaired proprioception are common symptoms of both DOMS [1,3] and ALS [4,5], as is found for other neurodegenerative diseases [6,7,8,9].

For a long time, the sensory deficit was not considered part of the symptoms of ALS, and it was viewed as a painless condition. Only recent research emphasizes the importance and dysfunction of the sensory circuits in ALS. Vaughan et al. [10] have noted in animal studies that significant structural changes are present in the sensory terminals of the muscle spindle before the onset of the symptoms of ALS, and these changes happen independently of the degeneration of α-motoneurons (α-MNs). Held et al. [11] even showed in an ALS mutant drosophila study that the sensory feedback circuits had been defective when the motor circuits were still intact. Brownstone et al. [12] proposed the microcircuit dysfunction theory in amyotrophic lateral sclerosis (ALS), which results in a runaway from homeostasis, eventually leading to MN dysfunction and death. According to a new theory [13], DOMS is an acute compression axonopathy at the proprioceptive sensory terminals in the muscle spindle. The contribution of these large fiber sensory afferents to DOMS was demonstrated in 2001 [14]. Correspondingly, both ALS and DOMS are proposed to have impairment at the proprioceptive sensory terminals in the muscle spindle. The present hypothesis highlights the analog homeostatic processes in DOMS as in ALS. Although the cause of force production loss is different, not surprisingly, the homeostatic compensatory processes are similar. DOMS is an acute and regenerating process, while ALS is a chronic and degenerating one with resultant sensory microcircuit dysfunction. The critical path of this ALS dying-back injury mechanism theory points toward the muscle spindle-derived sensory degeneration-induced non-resolving impairment of the proprioceptive circuitry.

## 2. Loss of Force Production Initiates the Homeostatic Process

The homeostatic mechanism keeps neurodegenerative diseases asymptomatic until a significant percentage of MNs are lost [12]. In some pools, even close to 70% of MNs degenerate before the onset of symptoms [15]. Degeneration progresses in an orderly fashion, with the largest α-MNs innervating the fast-twitch muscle fibers degenerating first, followed by the smaller α-MNs innervating slow-twitch muscle fibers [16,17].

The homeostatic faciliatory response to the loss of force production lies in the changes in circuits. Once the initial depletion of force-production activity of α-MNs on fast fatigable muscle fibers presents, the homeostatic response will be the premotor circuits increasing the firing rate of MNs, thus enhancing their excitation [18]. As a result, the output of the still-functioning α-MNs and γ-MNs, that are spared from degeneration [10], will be facilitated [19,20,21] in ALS.

The fatigue process prior to DOMS follows the order similar to the degeneration process of ALS, i.e., the largest α-MNs innervating the fast-twitch muscle fibers fatigue first, followed by smaller α-MNs innervating the slow-twitch muscle fibers. The consumption of ATP by slow-twitch muscle fibers is slower. Thus, fatigue resistance is higher in slow- than in fast-twitch muscle fibers [22]. The recruitment order of α-MNs follows the same orderly pattern that eventually leads to fatigue [23,24,25]. The force output of recruited α-MNs could be further modulated by increased firing, up to 8–10-fold [26]. Eventually, fatigue could reduce the firing rates of α-MNs by 30% compared to their initial values [27].

## 3. The Role of the Sensory Feedback Circuits and the Neuroinflammatory Link

The intrafusal muscle fibers contract out of proportion by γ-MNs due to the facilitation of the premotor circuits in ALS [12]. γ-MN activity is further enhanced when focusing attention on a task secondary to any perceived weakness present [28]. The facilitated premotor activity and the concomitant sensory afferent output from the muscle spindle will eventually end in an escape from homeostatic responses in ALS, which further enhances excitotoxicity [29]. Once the first symptoms appear in ALS, a portion of Type Ia/II sensory fibers are already worn out [10,30]. It is important to note that the sensory afferents from the muscle spindles provide direct innervation of MN pools, but ALS-resistant MN pools basically get no proprioceptive, monosynaptic feedback. The strength of heteronymous connections is weaker, in general, than the monosynaptic ones [12]. Therefore, proprioceptive impulses could be processed in a slower manner through heteronymous connections and force production is compromised when direct sensory innervation is impaired.

The author of this hypothesis highlights four major differences in ALS and DOMS regarding the pathology of Ia afferents in the muscle spindles. The first difference is the time dimension because ALS is a chronic, fatal degenerating disease, while DOMS is an acute, regenerating condition. The second difference is when the muscle spindles contract (ALS) [12] or stretch (DOMS) [13] out of proportion and the damaging superposition of compression forces are theorized to be the primary damaging forces in DOMS, whereas the Type Ia sensory terminals may detach from the intrafusal muscle fibers in ALS [10]. The third difference is the underlying systemic inflammation in ALS, as opposed to local inflammation in DOMS. Recent experimental and clinical observations point towards the fact that inflammation plays a more significant role in ALS pathogenesis [31]. The vast majority of ALS is late adult-onset, and Mészáros et al. [32] even suggest that neuroinflammation could come earlier than ALS symptoms. Finally, the central nervous system (CNS) involvement is dominant in ALS, while the peripheral nervous system (PNS) involvement is dominant in DOMS. While PNS has an impressive capability for regeneration, the CNS is incapable of full regeneration. Thus, we see a full functional regeneration in DOMS, but not in ALS.

It has been demonstrated in animal studies that even remote injury of peripheral nerves could result in impairment of the blood–spinal cord barrier (BSCB) functional and molecular integrity through a selective inflammatory pathway [33]. It is important to note that there is evidence for morphological and functional differences between BSCB and blood–brain barrier (BBB), and BSCB is suggested to be more permeable [34,35]. Based on the acute compression axonopathy theory of DOMS, the muscle spindle’s selective barrier permeability increases due to temporary mechanical and energy insult of the sensory axon’s terminal [13]. The signaling of this permeability increase is attributed to bradykinin of the COX-2-bradykinin-NGF axis [13,36,37]. The current author proposes that the degenerative proprioceptive process of the muscle spindles in ALS could result in an impaired muscle spindle selective barrier integrity or even impaired BSCB integrity at the early stage of the disease. The dysfunctional integrity of these selective barriers could provide the window of opportunity for local inflammatory signaling from the muscle spindle to propagate towards the CNS. There is evidence of compromised BSCB in early and late symptomatic SOD1 mice ALS models [38]. Furthermore, BSCB damage was presented in rodent and human ALS findings as well. Regardless, the pathogenesis of the BSCB impairment is far from clear [35,39,40]. Libby et al. [41] revealed, in ischemic cardiovascular diseases, that local and systemic inflammation could be linked by leukocytes. The total leukocyte count is elevated in ALS as well [42], and inflammation is present systemically. It is likely that the low-grade never resolving neuroinflammation of the aging process (inflammaging) [32], other than ALS associated genetic and environmental factors, further facilitates the degeneration of the peripheral proprioceptive terminals of the muscle spindles [43] and thus, creating a neuroinflammatory vicious circle in ALS. This non-resolving systemic neuroinflammation and the degeneration of proprioceptive sensory fibers in the presence of environmental and genetic factors lead to the imbalance of microcircuits and eventually a breakaway from homeostasis as suggested by Brownstone et al. [12].

Schram et al. [44] concluded in a review paper that a single nerve injury, even nerve crush injury, could initiate an earlier ALS disease onset and thus facilitate the local spread of the disease. They attributed the augmentation of inflammation to microglial cells. They were also capable of blocking the spread of the neuroinflammatory disease, fueled by neuron-glia signaling, with a result of increased survival. One of the signaling factors is neuregulin-1 (NRG-1), and the inhibition of it proved to be successful in the SOD1 G93A mouse model [44].

There are differences in nociception in ALS and DOMS as well. According to the acute compression axonopathy theory of DOMS [13], the micro-injured Type II sensory fibers become hyperexcited, causing the initiation of hyperalgesia with delayed onset. The micro-injured Type Ia fibers’ conduction velocity is decreasing with a delayed onset to a point where the conduction velocity of Type II fibers is higher. In ALS, the nociceptive impulses cannot come from the degenerating muscle spindles because the Type Ia/II fibers are degenerating together due to detachment [10]. Therefore, the suggested detachment is ‘silent’ and the nociception is primarily mediated through Type III/IV sensory fibers in ALS. It has been demonstrated that the combination of metabolites produced by exercise could activate Type III/IV sensory neurons resulting in fatigue and muscle pain [45]. The administration of these metabolites at low levels contributed to the perception of fatigue, while at high levels, it contributed to the perception of pain [45]. Certain metabolites could be elevated in ALS due to dysfunctional metabolism of the degenerating muscle fibers that could be an additional source of fatigue and pain. It has been shown that 66% of ALS patients experience pain, but only 9% suffered from a neuropathic type [46].

The orderly fashion of fine programming of the nervous system is manifest again because Type Ia sensory fibers are the fastest conducting fibers, followed by Type II, Type III and eventually the unmyelinated Type IV fibers, which are the slowest [47]. According to the gate control theory of pain [48], we could see a genuine velocity race of nociceptive transmission to the dorsal horn where Type II sensory fibers are suggested to arrive first in DOMS [13], while the Type IV sensory afferents arrive primarily after the concomitant degeneration of the Type Ia and II sensory fibers in ALS.

In summary, this dying-back injury model proposes that there is a primary phase (mostly silent) when the injury of peripheral sensory neurons of the muscle spindle and the resultant local neuroinflammation, in the form of a peripheral sensory axonopathy, link to a non-resolving systemic neuroinflammation of the CNS, namely, to inflammaging as a second phase. It is noteworthy that peripheral neuropathy could evolve as a combination of six mechanisms: altered metabolism, covalent modification, altered organelle function and reactive oxygen species formation, altered intracellular and inflammatory signaling, slowed axonal transport, and altered ion channel dynamics and expression [49,50]. It seems that the pathology of inflammaging must include the pathological mechanisms of the involved peripheral sensory neuropathy as a common denominator, and as a result, the non-resolving inflammaging entraps the local peripheral muscle spindle-derived proprioceptive axonopathy and leads to degeneration instead of regeneration. Microglial cells [44], which crosstalk with astrocytes [51], facilitate the spreading and augmentation of crosslinked local and non-resolving systemic neuroinflammation in ALS. Microcircuits, especially sensory microcircuits, try to maintain homeostasis, but the sensory axonopathy entrapped by non-resolving augmented neuroinflammation eventually leads to a breakdown of homeostasis instead of regeneration.

## 4. The Role of the Autonomic Nervous System

When a muscle is not capable of sufficient force production because it is fatigued, it is essential that the muscle activity should be maintained cognitively at the previously accustomed performance level than “over-reaching” is desired in order to accomplish. Coaches and athletes often use this “over-reaching” response as an objective in training (learning) sessions [52,53]. In fact, subsequent effective “over-reaching” training sessions, in combination with adequate recovery periods, could result in a higher level of homeostasis due to acute adaptation. This deviation from resting homeostasis is called super-compensation [54]. The “over-reaching” response and “supercompensation” are guided by the autonomic nervous system [53].

The activity of the autonomic nervous system could provide the helping hand necessary for “over-reaching” in the form of an acute stress response (ASR) [13]. ASR has two dimensions: “heat of battle” response and “fight or flight” response. During the “fight or flight” response, the increased sympathetic loading dampens the feedback control of muscle length [55,56,57,58]. The low feedback control is beneficial in ASR because fine movements can be traded for “fight or flight responses” [55]. During a “heat of battle” response, sympathetic nervous system (SNS) activity suppresses pain by descending inhibition of nociception in the spinal cord [59], and the faster conducting non-nociceptive Type Ia sensory fibers indirectly inhibit the effects of the nociceptive Type II sensory fibers. When the force production is unacceptably insufficient, then cognitive demand induced ASR (“over-reaching”) could prevail as a driver, and the sensory afferent terminals of the muscle spindle could be micro-damaged by repetitive compressive eccentric contractions. This will result in a relatively higher conduction velocity of nociceptive Type II sensory fibers with a delayed onset of pain [13]. Therefore “over-reaching” is provided without the immediate limitation of pain sensation.

Due to the gradual loss of MNs in ALS, there is a point when functioning α-MNs cannot be modulated for the loss of others. This must be a point in ALS disease progression that ASR is induced on a frequent basis because underperformance of muscle activity is not accepted by cognitive demand, and “over-reaching” is needed to accomplish the expected result. In animal models, there is evidence that ALS-inducing factors directly affect and sensitize sensory neurons to stress [60]. There is also evidence of hyperactivity of the autonomic nerves as an early feature in ALS, with further evidence of hypoactivity as the disease progresses [61]. The hypoactivity of the autonomic nerves could be translated as the depletion of the “over-reaching” process. It also has been suggested that the stability of cholinergic synapses beyond proprioceptive synapsis is impaired and affects the neuromuscular junctions even at the early presymptomatic stage of ALS [62].

It is unlikely that MNs are automatically capable of “over-reaching” by increasing their firing rate beyond their normal limits. Therefore, the circuit function could drive homeostatic response by increasing excitation of MNs [12]. The author of this paper proposes that ASR could be such a homeostatic driver as well, conducted by the direct sympathetic innervation of the muscle spindles [55].

## 5. The Role of Impaired Proprioception and the Dying-Back Injury Model

Following DOMS initiation, there is a reduced range of motion [1]. Even though there is some compensatory mechanism in the DOMS-affected muscles, the reduced range of motion also comes with impaired ability to attenuate shock. It has been demonstrated in animal studies that there is increased attenuation of shock in the DOMS-affected muscles, but concomitant reduced attenuation at the head of the animal [63]. These findings could be translated to explain why the expense of local proprioceptive overcompensation of micro-injured sensory axon terminals (DOMS-affected muscles) is so high that the proprioception of the neck area suffers in this abrupt neuro-energetic resource reallocation process. Therefore, the impaired proprioception and the resultant impaired kinematics under ASR and DOMS could result in a secondary, more damaging whiplash-like injury. The mechanism is in line with the dichotomous acute compression sensory axonopathy theory of DOMS [13], where the secondary injury phase is even more damaging [13,64,65]. The proposed phenomenon is even more detrimental, along with proprioceptively impaired ALS disease progression, due to the muscle spindle and muscle wasting feature of the disease, because shock attenuation is progressively diminished.

In addition, the author is suggesting that the increasingly impaired integrity of the BSCB and BBB also have relevance in this phenomenon with ALS disease progression. This suggestion provides increased access to potentially micro-damaging antidromic waves and external physical impacts. In support of this theory, there is evidence that short- burst, phase keying focused ultrasound could mitigate physical waves that could open BSCB without bleeding at low pressures and with widespread focal bleeding at higher pressures [66]. It is noteworthy that in ALS, the amplitude of the largest antidromic F-waves is significantly higher than in healthy subjects. These giant F-waves are attributed to newly formed distal axonal branching [50]. These antidromic giant F-waves could sustain the impairment of the BSCB and, as a result, provide the window of opportunity for further micro-damage to the spinal cord by antidromic waves, eccentric contractions and physical impacts.

On the other hand, orthodromic waves could have relevance in the hyperexcited gamma loop of the muscle spindle (the equivalent of the muscle spindle contracting out of proportion as Browstone proposed [12]) because hyperexcited γ-MNs hyper-excite the sensory neurons in ALS. Sábado et al. [67] reported in an animal study that large proprioceptive neurons accumulated misfolded SOD1 in the dorsal root ganglion (DRG) and went through a degenerative and inflammatory process. This could mean that the misfolded SOD1 in the large proprioceptive neurons are the result of the hyperexcitation of these sensory neurons to a level that causes intracellular micro-damage and neuroinflammation. It is even more speculative to propose that the unfolding proteins in neurodegenerative diseases are not accidental, but they are initially part of a protective mechanism to provide physical counter support to these non-resolving, micro-damaging physical waves and impacts. Although over time, the morphological manifestation of these unfolding proteins could eventually cause functional problems in neurodegenerative diseases depending on the location of accumulation, and that could further exacerbate disease progression. Noteworthy that resistance exercise (dominantly contain eccentric contractions) is inducing unfolded protein response (UPR) [68]. UPR is increasing when there is unfolded protein accumulation. The author of this article is proposing that in DOMS, the UPR pathway is activated in a normal way. Therefore, full functional regeneration will follow in the proprioceptive sensory neurons of the muscle spindle. On the other hand, the UPR pathway is in a state of imbalance in ALS. Therefore, it facilitates constant inflammation, degeneration and eventually apoptosis. Furthermore, Sábado et al. [67] showed in the same animal study that the degenerating sensory axons were associated with activated microglial cells in the dorsal horn of the spinal cord. This observation could provide an explanation of how microglial cells play a nonunivocal role during stress and injuries [69] and propagates neuroinflammation in the CNS of ALS patients [44].

Sábado et al. [67] interpreted their findings as the SOD1 misfolding mechanism originates from the ventral horn MNs and, in a prion-like manner, eventually micro-damages the DRG sensory neurons. It is even more likely that both orthodromic and antidromic waves do so due to the hyper-excited sensory- and motor neurons. Glutamate has been detected in motor neurons and in peripheral sensory neurons as well, and electrical or chemical stimulation can release it [70]. The Type Ia sensory fibers could be sensitized by glutamate [71] and mediate fast glutamatergic signaling, but glutamate could also provide the retrograde neurotransmitter signaling in the motor neurons as well [70]. Glutamate is a central player in pathological conditions of the CNS, such as ischemia, inflammation, traumatic injuries, and mental disorders [70,72]. Glutamate excitotoxicity has been suspected for a long time in the pathogenesis of ALS [73].

Physical injury and stress are often implicated as risk factors in neurodegenerative diseases. Proske and Gandevia [74] have implicated that damaging eccentric exercise is responsible for the impairment of proprioception. Eccentric contractions are under the neural control of the Type Ia and Type II sensory neuron in the muscle spindle. As the difficulty of task execution increases under enhanced cognitive demand, with the assistance of the autonomic nervous system, then induces more eccentric contractions in order to convey high force generation to assist acceleration and deceleration movements. Eccentric contractions are using higher cortical excitation and lower motor unit discharge [64,75]. Furthermore, eccentric contractions also have the characteristics of absorbing energy from an external load [76], supporting the body against gravity, absorbing shock, and storing recoil energy for accelerating contractions [64,77]. Eccentric contractions were named as negative muscular work by Abbott et al. [76]. Nonetheless, there is no such thing as negative work according to physics, but eccentric contractions pertain to the storing of recoil energy that could come from, e.g., ground reaction forces (that is, the force carried out by the ground as a reaction to the forces a body applies on it [78]). The problem arises when the storing of energy from the external load, coming from the accelerating movement, cannot “recoil” in the decelerating movement. The author of this paper is proposing that the excess “unrecoiled” energy coming from accelerating movements is partially engulfed by the PNS and CNS with concomitant compression in a damaging way when the selective protective barriers are impaired, like muscle spindle selective barrier, BSCB and BBB. Thus, the notion that peripheral proprioceptive sensory axon injuries under ASR have impaired shock attenuation and impaired proprioceptive capabilities could have implications for contact injuries, DOMS, noncontact injuries, whiplash injuries and mild traumatic brain injuries because they may not disappear without a PNS and CNS footprint. It is important to note that muscles and other tissues also absorb excess “unrecoiled” energy from eccentric contractions in a damaging manner.

Overall, eccentric contractions, cognitive demand, fatigue, and stress are essential contributors, both acutely and chronically, to peripheral sensory neuronal injury and degeneration in this theoretical ALS dying-back injury model. Furthermore, the imbalance of the adequate modulation of the bidirectional crosstalk between the autonomic and proprioceptive sensory nervous system will lead to accelerated ALS disease progression.

## 6. COX-1, COX-2, PGE2, GDNF, NGF and Glutamate Signaling Pathways

Inhibition of COX-2 improves motor strength and survival in ALS transgenic mice at the spinal level [79]. This inhibition shows neuroprotective effects in ALS and neurodegenerative diseases [80], while dual-action COX-2 inhibitors prevent DOMS [81]. Mizumura et al. [37] attributed the COX-2-PGE2-GDNF pathway to Type III sensory fibers’, and Murase et al. [36,82] attributed the COX-2-bradykinin-NGF pathway to Type IV sensory fibers’ sensitization. The acute compression axonopathy theory of DOMS [13] attributed the COX-1-PGE2 pathway to the hyper-excitation of Type II fibers, based on the work of Sun et al. [83]. Finally, Type Ia sensory fibers have been suggested to be sensitized by glutamate [13,71,84,85]. COX-1 plays a neuroinflammatory damaging role in the CNS, while COX-2 has a pro-inflammatory effect on the periphery, and it is rather neuroprotective in the CNS [86]. COX-2 is present in spinal neurons and astrocytes. PGE2 increases glutamate release from astrocytes, and glutamate excitotoxicity is implicated in ALS [79]. Astrocytes normally have a role in synapse formation, propagation of action potentials, maintenance of extracellular matrix homeostasis, and more important, maintenance of BSCB and BBB [87]. Furthermore, astrocytes are constantly engaged in intimate molecular crosstalk with microglial cells. Microglial cells are responsible for restoring homeostasis in the micro-environment of the CNS and serve as the first line of immune cells in the CNS. During CNS insults and injuries, microglia-astrocyte molecular conversation, regulated by microglial cells, has immense relevance [51]. Microglial cells are thought to be responsible for the augmented propagation of neuroinflammation in ALS [44].

The satellite cell, an astrocyte-like stem cell, is thought to have a functional role in muscle plasticity. Tamura et al. [88,89,90] noted in animal studies that aerobic exercise increased the number of satellite cells, but the proliferation was uncoupled from fusion. When the depletion of satellite cells in wheel running was investigated in genetically altered mice (Pax7/DTA) with tamoxifen administration, it was found that voluntary running performance was decreased and proprioception was impaired, possibly due to disruption of muscle spindle fibers [91]. Interestingly, a higher number of satellite cells were found in the intrafusal muscle fibers than in extrafusal fibers [92]. Furthermore, satellite cell depletion enhanced the atrophy of the muscles and caused disruption at the neuromuscular junction [93].

Li [94] and Suzuki et al. [95] have demonstrated that the intramuscular administration of muscle (not centrally)-derived GDNF resulted in a higher number of preserved innervated neuromuscular junctions and surviving MNs, even at later stages of ALS. Thus, it seems that the excitation of the COX-2-PGE2-GDNF pathway is enhancing the neuromuscular junction stability at the spinal and peripheral levels of the nervous system.

If we try to translate these findings through the acute compression sensory axonopathy theory of DOMS [13], then the uncoupling effect of aerobic exercise could be contributed to the fatiguing mechanism under the intensified neural control of Type III/IV sensory nerve pathway over the Type Ia/II sensory fiber’ pathway. In aging and in neurodegenerative diseases, the dominance of Type III/IV sensory nerve control could have similar effects because there is a more significant disruption of the neuromuscular junctions in the muscle spindle than in the extrafusal neuromuscular junctions. An explanation for this observation could be that the more sophisticated and energized compartments are likely to be affected first in the degeneration process.

If the loading of Type Ia/II sensory fibers gain dominance in neural control by eccentric exercise, then the author suspects that the coupling of proliferation and fusion of satellite cells could be enhanced. Furthermore, using the “open gate exercise” [13] protocol, meaning high-intensity DOMS-inducing eccentric exercise, the intrafusal satellite cells could contribute to the increased permeability of the selective barrier of the muscle spindle due to the suggested COX-1-PGE2 pathway providing access for intra-extrafusal crosstalking at the PGE2 level. Since, both in ALS and DOMS, compression proprioceptive axonopathy is suspected, it is noteworthy that at the early stage of peripheral nerve injury, astrocytic responses are possibly serving the purpose of nerve regeneration and motor functional recovery [96], could also increase BSCB permeability, in order to provide access for crosstalking of pathways at the COX2 and PGE2 levels between muscle fibers, muscle spindles, PNS and CNS.

In acute injury models, such as cerebral ischemia and seizures, COX-2 promotes neuronal injury [97]. In addition, in chronic inflammation and neurodegeneration models, like in Alzheimer’s disease, Parkinson’s disease, and ALS, COX-2 enhances inflammatory injury. PGE2 signaling on EP2 receptors in the CNS has a dichotomous action depending on whether the injury is acute excitotoxicity or chronic inflammatory. In acute cerebral ischemia and excitotoxicity, the EP2 signaling is conveying enhanced neuroprotection. While in chronic inflammation and neurodegeneration models, the EP2 signaling may result in secondary excitotoxicity [97]. The author claims that DOMS belongs to the acute injury model.

## 7. What We Can Learn from Axonopathy-Causing Chemotherapy

Bennet et al. [98] hypothesized that the axon terminals with the highest energetic demand could go under terminal arbor degeneration (TAD) due to impairment of the energy supply of the mitochondria in an energy-demanding environment. It has been known that the TAD mechanism could be induced by axonopathy-causing chemotherapy, like with paclitaxel and oxaliplatin [49,98]. Bennet et al. [98] demonstrated, with paclitaxel, that the surfacing of symptoms was threshold driven by accumulating toxicity and was dosage-dependent. Paclitaxel evoked TAD with neuropathic symptoms at low-dose thresholds without axonal degeneration. At a higher-dose axonal degeneration could be observed, while an even higher-dose threshold could cause apoptosis of the sensory neurons.

It is notable that a study has been launched to prevent oxaliplatin-induced peripheral neuropathy by Riluzole [99]. Until recently, Riluzole was the only approved life-lengthening drug for the treatment of ALS [100]. In animal studies, Riluzole prevented glutamate excitotoxicity when they were treated with oxaliplatin, as was the case in ischemic animals [99,101,102,103,104]. The neuroprotection is eminent in the brain, spinal cord and retinal ischemia through this effect [99,101,102,105]. Furthermore, Riluzole improved the neurological status of patients with spinal cord injury and did so without significant side effects [99,106]. There is a therapeutic need to reduce or prevent oxaliplatin-induced peripheral neuropathy in chemotherapy, and it seems to be the rationale that Riluzole could provide this beneficial property.

Bullinger et al. [107] demonstrated in a study with rats that even the non-degenerated axons have proprioceptive impairment after oxaliplatin treatment. Furthermore, Vincent et al. [108] showed a novel chronic proprioceptive impairment with oxaliplatin chemotherapy. Both of these studies are pointing toward the dysfunctional encoding of the sensory fibers of the muscle spindles. Wang et al. [109] not even showed proprioceptive deficits in cancer survivor patients but also demonstrated they are associated with motor dysfunction. Vincent et al. [108] suggested that oxaliplatin chemotherapy could lead to movement disability by impairing sensory encoding. They hypothesize that oxaliplatin exerts its neurotoxic effect on persistent inward sodium currents (NaPIC) through the micro-damaged sensory terminals of muscle spindles. NaPIC is responsible for sustaining repetitive firing during static muscle stimulation. It is noteworthy that the encoding of the dynamic changes in muscle length was barely affected [108]. Riluzole, among other drug action properties, has its only antagonist action on NaPIC. Therefore, the administration of Riluzole results in a selective modification of proprioceptive signaling in oxaliplatin-treated rats.

In summary, oxaliplatin treatment could cause peripheral sensory axonopathy and the impairment of proprioception in a dose-dependent manner. Chronic administration of oxaliplatin could even result in movement disability. Riluzole, a life-lengthening ALS drug, demonstrated to be beneficial in animal studies to treat oxaliplatin-induced peripheral axonopathy and expected to be so in human studies. Riluzole has multiple drug actions and certainly has beneficial properties to prevent glutamate excitotoxicity, reduce peripheral neuropathy and enhance proprioception. The author of this paper is proposing that chronic oxaliplatin chemotherapy administration highlights a chronic proprioceptive sensory impairment pathway that has relevance in ALS disease progression and movement disability mechanisms. Noteworthy again that oxaliplatin and paclitaxel at low doses could cause peripheral sensory axonopathy without degeneration, and this type of non-degenerating axonal microinjury is suspected in the compression sensory axonopathy of DOMS theory [13] as well. Impairment of proprioception is evident in ALS, DOMS and oxaliplatin treatment that implies the critical involvement of muscle spindles.

## 8. Repeated Bout Effect of DOMS May Offer an Explanation for the Longitudinal Dimension of ALS

An exercise bout is no longer unaccustomed after an initial bout of severe DOMS-inducing unaccustomed exercise entailing eccentric contractions. It was postulated that the exercised muscles protect themselves from further damage, therefore repeated bout of similar eccentric exercise would be less damaging. This phenomenon has been named the repeated bout effect (RBE) by McHugh [110]. The exact mechanism of RBE is not known, and several theories are running. Impaired proprioception is comprised in the definition of DOMS [1], but it is an often forgotten symptom. This current hypothesis is highlighting the relevance of impaired proprioception in RBE.

Glutamate is a fast signaling neurotransmitter in the nervous system. The Type Ia sensory fibers have been proposed to be sensitized by glutamate under pathological conditions [13,71,84,85]. Repetitive eccentric contractions under ASR are suggested to be such a pathological condition [13]. This pathological condition is called glutamate excitotoxicity and proposed at the axon terminals of the primary sensory afferents of the muscle spindle in DOMS [13]. There are several suggested consequences at the central presynaptic end at the dorsal horn of the hyper-excited sensory afferents due to this glutamate excitotoxicity, like gate control, substance P release, NaPIC amplitude increasing activity and astrocyte-to-neuron signaling.

N-methyl-D-aspartate (NMDA) receptors at the presynaptic end of the pseudounipolar large fiber sensory afferents, and not on the DRG cell bodies, could be responsible for the complex activity-dependent control of glutamate release [111]. Russo et al. [112] showed that glutamate excitotoxicity could activate presynaptic NMDA receptors (NMDAR) endogenously [111]. The glutamate spillover and the activation of the sensory throughput regulating feature of this NMDAR are suggested by the author of this article to lead to the acute compression axonopathy of the primary afferents of the muscle spindle in DOMS and ALS. It is noteworthy that Murase et al. [36] have suspected the involvement of NMDA receptors in DOMS.

The glutamate excitotoxicity of DOMS is proposed to happen under an osteocalcin-induced ASR [13]. Osteocalcin is a fast, bone-derived stress signaling protein hormone [113] that could penetrate the BBB [114]. This BBB penetrating feature of osteocalcin could be responsible for inducing descending inhibitory effect in the CNS that extends all the way to the spinal dorsal horn. It is speculative, but we should not exclude that osteocalcin may penetrate the BSCB as well and directly exerts stress-induced analgesia on the dorsal horn. Furthermore, osteocalcin exhibits a strong impact on spatial learning and memory as well [115], providing the basis for the RBE. Once the effect of circulating osteocalcin is diminishing, the parasympathetic tone is recovering in a gradually opposing way to the sympathetic tone and the proprioceptive sensory NMDARs are activated. This point is the end of stress-induced analgesia on the dorsal horn and the initiation of pain sensation in DOMS. It is noteworthy that cardiac autonomic full recovery measured by heart rate variability takes 24–48 h [53] and overlaps the peak of the ascending phase of hyperalgesia in DOMS [116].

The activation of the NMDARs at the central ends of the primary sensory afferents could interfere with the propagation of the action potentials along the presynaptic axon of the primary afferents [111]. This significant synaptic latency causing character of activated NMDARs [111] is suggested to be responsible for the delayed Type Ia sensory transmission, and as a result, the action potential of Type II sensory fibers is propagated earlier to the “gate” [13]. In addition, the activated NMDARs in animal models are suggested to release substance P from the central terminals of nociceptors at the dorsal horn [111]. This mechanism theory is in line with the gate control theory of pain [48] and the new acute compression axonopathy theory of DOMS [13]. In other words, the endogenously activated NMDARs are proposed by the current author to be responsible for the control and modulation of the “gate” in DOMS.

The monosynaptic and polysynaptic throughput is suggested by the author of this article to also be altered by the activated NMDAR in the fusimotor reflex arc of the axonopathy affected sensory fibers. Bardoni et al. [111] theorized that NMDAR, activated by repetitive hyper-stimulation, are proposed to activate a second-messenger pathway [111]. The amplitude of motoneuronal self-sustained persistent inward sodium current (NaPIC) is suggested to be increased due to the activated NMDAR. This could be an analog phenomena, like the observation that after nerve crush, the MNs show supranormal output regardless of sensory deficit [48]. Furthermore, the dendrites of MNs are capable of generating persistent inward currents (PIC) that could be an explanation for this supranormal MN gain [117]. Goff et al. [118] demonstrated that there is increased attenuation of shock at the DOMS-affected leg muscles, but reduced attenuation of shock at the head after DOMS initiation. These findings could be translated as damaging unaccustomed or strenuous eccentric contractions are generating motoneuronal persistent inward currents in the DOMS-affected muscles in order to attenuate shock and protect against gravity and enhance postural control at the segmental level of the acute proprioceptive axonopathy. This proprioceptive protection lasted 24–120 h [118]. It is noteworthy that this time interval overlaps with the delayed time frame of pain sensation in DOMS [37,116] and the time frame of the transient BBB and BSCB selective permeability in peripheral nerve injury [33]. Furthermore, this supranormal proprioceptive protection at the DOMS-affected area comes at the price of impaired overall proprioception and impaired postural control of the body. This implies that the neuro-energetic resources of the proprioceptive system are limited.

Bullinger et al. [107] demonstrated in oxaliplatin chemotherapy-treated rats that even the non-degenerated sensory axons exhibit proprioceptive impairment. Furthermore, Vincent et al. [108] showed that oxaliplatin treatment has a chronic dimension to proprioceptive impairment. They also hypothesized that oxaliplatin exerts its neurotoxic effect on NaPIC of muscle proprioceptors [108]. Noteworthy again that the encoding of the dynamic changes component of the sensory impulses was barely affected [108]. Both Type Ia and Type II sensory fibers are encoding static components, but only the Type Ia afferent transcends dynamic changes components. The author of this article is proposing based on the above findings that the dynamic changes component of the proprioceptive Type Ia sensory information is also barely affected in DOMS, and this is why the short- and long-latency stretch reflex components are unaffected in DOMS [119]. On the contrary, the static component of the proprioceptive Type Ia sensory impulses are delayed by activated NMDAR and, as a result, diverted to induce compensatory microcircuits. These compensatory microcircuits are proposed to be the equivalent of the “second-messenger pathway” suggested by Bardoni et al. [111]. It is important to note that the microcircuits are less efficient and energy-consuming because the signaling involves more synapses. The activated NMDAR delayed static Type Ia sensory input also results in the earlier arrival of Type II impulses to the “gate”. Therefore, a portion of the monosynaptic static and adapting Type Ia sensory impulses are exchanged for non-adapting, constantly firing static Type II sensory encoding on the MNs. This exchange of monosynaptic static sensory input is suggested to increase the amplitude of NaPICs on the dendrites of MNs, which is the equivalent of the supranormal protection against gravity.

Hamilton et al. [63] demonstrated that there is a reduced range of motion after DOMS initiation in the affected muscles. It was theorized that the reduced range of motion is a compensatory protective measure [63]. These induced polysynaptic “second-messenger signaling pathways” [111] or protective microcircuits are proposed by this author to be the cause of a reduced range of motion in DOMS and is responsible for the enhanced postural control at the segmental level. Furthermore, the current author is proposing that the degree of the compression microinjury (extending from distal to central) of the annulospiral Type Ia sensory terminal correlates with the magnitude of glutamate spillover in a dose-dependent manner and could determine the extent of the reduced range of motion. Thus, the repetitive, excessive stretch of the muscle spindles due to its spindle shape means that the more distal or polar the microinjury of the annulospiral ending (or spiral ending in humans [120]), the less limitation could be on the range of motion [13]. Furthermore, the repetitive eccentric contractions induced extra-stretch along the flower spray [120] ending (or spiral ending in humans [120]), that is anchoring the Type II terminal to the intrafusal muscles, also causes compression microinjury and dose-dependent hyperexcitation on the nociceptive encoding [13] proportional to stretch. This could be the detailed explanation for the length dependence of DOMS and the absence of pain at rest [13].

The author of this article further proposes that a burst, single eccentric-derived excessive stretch of the muscle spindle without ASR causes strain injuries. This is why strain injuries are occurring at the beginning of training or task execution. The pain is immediately felt in this type of injury because there is no osteocalcin-derived descending inhibition. Worth to mention here that both repetitive and single burst eccentric contraction-induced proprioceptive sensory terminal microinjury could be progressively detrimental in ALS due to the proposed detachment from the intrafusal neuromuscular junctions [60].

The above invoked protective microcircuits and increased amplitude NaPICs are suggested by this author to interfere with the preprogrammed postural control of the body. In summary, there is enhanced postural control (static Type Ia sensory encoding) and enhanced protection against gravity (static Type II sensory encoding) of the body at the segmental level of the acute proprioceptive sensory axonopathy, but the loss of postural control and protection against gravity in the unaffected segments. This impairment of proprioception is suggested to be the result of neuro-energetic resource limitation in the proprioceptive system. The polysynaptic signaling of microcircuits are less efficient and highly energy demanding, therefore instigate an energetic resource reallocation in the proprioceptive system and the area will be affected the most that have the highest energy demand among the spinal segments without axonopathy (that is, the head–neck region [118]). Recovery of the preprogrammed postural control takes 24–120 h [118] after the initial bout of DOMS-inducing exercise. Thus, we could conclude that the recovery time of the neuro-energetic allocation system of proprioception overlaps the time frame of the functional regeneration of the micro-injured intrafusal sensory terminals. The central mechanism of the recovery of the preprogrammed postural control also explains the bilateral sensory deficit [3] and the homologous muscle involvement of the contralateral limb in DOMS [121,122]. Not to mention that the impaired proprioception also means disturbed position sense in DOMS [123].

The functional plasticity of the compensatory or protective microcircuits of DOMS could be evoked almost up to a year [124] with the same or near same exercise bouts as the initial one. It also means that RBE is almost up to one year remembering how to restitute the preprogrammed postural control of an acute large fiber sensory axonopathy affected spinal segment under an enhanced protective anti-gravity measure. This implies that DOMS and RBE have several memory and learning dimensions. Osteocalcin and activated NMDA play an important role in the initiation and controlling of synaptic plasticity, memory and learning [115,125]. The mapping of these pathways is not the task of this manuscript, but it seems that the activated NMDARs of proprioceptive sensory central terminals on the dorsal horn are providing the “gate control” of the peripheral encoding for temporal and spatial summation. It also seems likely that short-term working memory, long-term episodic memory, inflammation memory and pain memory are affected by activated NMDARs in DOMS and RBE. Noteworthy again that the acute stress signaling osteocalcin has a strong impact on memory and spatial learning as well [115]. Activated NMDARs on astrocytes could be the critical novel pathway that leads to astrocyte-to-neuron signaling [126]. Glial cells also go through stress-induced changes and contribute to learning and memory [127]. Not to mention its role in the augmentation of neuroinflammation in ALS [44].

The current author is proposing that, in ALS, the proprioceptive axonopathy-induced realignment process of the postural control cannot be completed by compensatory microcircuits, like it is suggested in DOMS and RBE, due to the “silent” detachment of the sensory terminals. The progressive “silent” loss of proprioceptive sensory terminals in ALS could lead to the progressive and non-resolving dysfunction of microcircuits. Since an important part of position sense encoding is derived from the muscle spindles [74], the spatial summation of short-term adaptation and long-term learning (the equivalent of RBE) are both suggested to be compromised in a non-resolving way in ALS. Furthermore, the dysfunctional microcircuits eventually interfere with the motor command and the locomotor circuits. Thus the loss of plasticity will not allow locomotor recovery. The progressive and excessive neuro-energetic demand of the dysfunctional microcircuits in ALS could be deduced from the proposed DOMS and RBE mechanism.

Worth to mention, that another fatal consequence of the dysfunctional microcircuits could be the progressively dysfunctional synchronization/harmonization of the life-sustaining central pattern generators (CPG) due to the increasing number of disturbingly high amplitude PICs [128].

## 9. Precise Targeting with Exercise Intervention

Brownstone et al. [12] proposed that targeting microcircuit dysfunction should help postpone the progression of ALS, meaning reduction of γ-MNs activity or increased activity of Renshaw cells. The question arises whether this strategy could be implemented by exercise intervention.

Tsitkanpu et al. [129] noted that mild-to-moderate intensity exercise, especially swimming, could increase survival and postpone the onset and the progression of ALS in SOD1^G93A^ mice models [130,131]. Even though clinical studies showed positive benefits of exercise on the quality of life of ALS patients, they failed to demonstrate life extension [129,132].

In order to target sensory microcircuit dysfunction, the loading of the muscle spindle should be minimized during exercise intervention. Different exercise and contraction types have different characteristics. For example, the neural control of eccentric versus concentric and isometric contractions is considerably different [133]. Eccentric contractions have better force generation [134] and neuro-energetic profile [76] than concentric contractions. On the other hand, the inflammatory characteristics of concentric exercise are better when intensity increases. In fact, eccentric exercise, especially if unaccustomed at high-intensity, is considered to be damaging to muscle [135]. However, the damage does not stop at the muscle level. Other tissues could be affected, such as connective tissue, extracellular matrix [136], or even the terminals of the sensory afferents [13]. The positive neuro-energetics of eccentric contractions likely come at the price of increased proprioception and a resultant heavier load on the sensory afferents in the muscle spindle. The muscle spindle could contract out of proportion in ALS [12] and stretch out of proportion during eccentric contractions [13]. These two opposing forces would impose additional stress on the muscle spindles and greater risk because the Type Ia/II sensory terminals could detach from the intrafusal muscle fibers due to the lack of intrafusal muscle-derived signaling in ALS [10]. Evidently, eccentric exercise should not be the choice of exercise modality in ALS or other muscle spindle-affected neurodegenerative diseases.

High-intensity exercise and fatigue should also be avoided because, over time, when intensity is increasing, the number of eccentric contractions and the loading of muscle spindles is increasing and could potentially induce DOMS. According to the acute compression axonopathy theory [13], DOMS means an “open gate” in terms of the gate control theory of pain [48]. These authors also noted that exercise at “open gate” could have unfavorable systemic inflammation enhancing profiles in degenerative diseases [13], such as neurodegenerative diseases. Part of the acute compression axonopathy theory of DOMS that “open gate” is also accompanied by increased muscle spindle selective barriers and possibly BSCB permeability, thus providing greater access to the PNS and CNS and therefore, a linkage to systemic pro-inflammatory signaling [33,137]. Based on this theory, “closed gate exercise” [13] would be the proposed exercise of choice in neurodegenerative diseases due to the already present systemic inflammation, which means light-to-moderate exercise.

Zainuddin et al. [138] showed that light concentric exercise has a temporary analgesic effect on DOMS but has no impact on the regeneration from muscle damage. The acute compression axonopathy of DOMS theory could explain the temporary analgesic effect because when light concentric exercise is executed, then the micro-injured Type II nociceptive fibers are not excited, and thus, the excited Type III sensory fibers are bypassing the conduction velocity of the Type II fibers. When exercise is finished, the conduction velocity of Type III sensory fibers drops below the conduction velocity of the micro-injured Type II nociceptive fibers, and therefore, pain comes into play again. This concept is analogous with the clinical findings of the positive benefit of exercise on the quality of life of ALS patients, but the investigators failed to demonstrate any prolongation of the life span [129,132]. The explanation behind this could be the analog homeostatic process.

In ALS, light concentric exercise could mean that the conduction velocity of Type III/IV sensory neurons is bypassing even the still existing Type Ia/II sensory neurons’ conduction velocity. Moreover, at high intensity, the combination of metabolites produced by exercise could activate the nociceptive Type IV sensory neurons [45]. Thus, high-intensity concentric exercise should be also avoided in ALS, because once muscle starts to degenerate, i.e., with disease progression, the metabolites increase. This could also excite nociceptive Type IV sensory afferents, and at this stage of the disease, it is likely that the BSCB and BBB permeability are also significantly increased, and neuroinflammation could be further augmented.

It is also worth mentioning again that muscle-derived, and not centrally derived, GDNF could preserve the neuromuscular junctions and increase the number of surviving MNs even at the mid-to-late-stages of ALS disease progression [94,95]. The COX-2–PGE2–GDNF pathway is attributed to Type III sensory fibers excitation [37]. These findings further substantiate the recommended strategy of light concentric exercise, which dominantly keeps the exercise under Type III sensory fiber neural control.

Lalancette-Herbert et al. [20] suggested limiting the fusimotor drive, not just as a symptomatic benefit, but possibly to lengthen the life span with a higher quality of life in ALS by delaying lower MN degeneration, especially in those with predominant upper MN findings. Closed kinetic chain exercise, like stationary bike cycling, may possibly further unload the proprioception in the muscle spindles. Pedaling in cycling probably causes a higher shear force and loading of proprioception within the affected muscle spindles than does swimming, but not as much as open kinetic chain exercise. Yack et al. [131] demonstrated first that there was greater joint laxity (probably greater muscle laxity as well) during open kinetic chain exercise [139]. Closed kinetic chain concentric stationary cycling (proprioception constrained to only two dimensions, meaning less laxity), as opposed to outdoor cycling exercise (three-dimensional proprioception, meaning greater laxity), could reduce this shear force (reduced mechanoreceptor stimulation) in addition to unloading of proprioception. It is important to note that the difference in proprioception input lies in dimensional differences because exercise in three dimensions certainly refers to enhanced position sense. Position-sense-loading implies considerable load on the CNS [140], which is costly in terms of neuro-energetics. The author of this paper suggests that cleats should be used in order to minimize position sense by constraining the ankles. Thereby further unloading proprioception. Unloading proprioception and the resultant circumvention of central sensory-loading with “closed gate” brings into account closed kinetic chain concentric exercise, like stationary bike cycling with cleats or even better with electric assistance. The signaling basis for this strategy could be the finding that NMDA could actively produce intrinsic rhythmic activity, along with the central pattern generators, in MNs of adult rodent in order to produce swimming-like locomotion [141]. With this strategy of exercise therapy, one could circumvent the fusimotor drive as much as possible, as Lalancette-Herbert et al. [20] proposed. Furthermore, one could avoid muscle and neural-damaging exercise, unload proprioception in order to be neuroprotective and anti-inflammatory. One could enhance cellular tight junctions, possibly decrease selective barrier permeability, possibly enhance the plasticity of glial-, neuromuscular junctions, and neuroplasticity.

## 10. Conclusions

In this paper, the author has hypothesized the analog homeostatic processes between DOMS and ALS. Acute compression sensory axonopathy, caused by eccentric contractions under ASR, induced microcircuit dysfunction, environmental and genetic factors and an escape from homeostasis mean eventually a point of no return in the case of muscle-spindle-affected neurodegenerative diseases, due to the “silent” detachment of peripheral sensory nerves in the muscle spindle, while regeneration and functional restitution prevail in DOMS. Highly important to note in order to avoid misinterpretation that there is no direct link between DOMS and ALS. The parallel is drawn in terms of the homeostatic processes. Furthermore, the sensory deficits are not dominating the clinical picture of ALS, and the current emphasis is constrained only to muscle spindle related sensory impairments.

The acute compression axonopathy theory of DOMS entails both neuronal injury and stress as they are often implicated in neurodegenerative diseases. Furthermore, ASR is suggested to be a homeostatic driver in the “over-reaching” response when force production is depleted, but the performance should be maintained in order to accomplish this. DOMS and the RBE could lead us to a better understanding of the longitudinal axis of the time dimension of neurodegenerative disease progression. The recommended exercise of choice in ALS and other muscle-spindle-affected neurodegenerative diseases is to circumvent the fusimotor drive as much as possible and “closed gate”. Light closed-kinetic-chain concentric exercise like stationary bike cycling with cleats or even better with electric assistance seems to be a good choice.
